# Cannabis Use and Cardiovascular Risk Among Healthy Young Adults: Cause or Confounding?

**DOI:** 10.1016/j.jacadv.2025.101961

**Published:** 2025-07-14

**Authors:** Adam Cardone, Arman A. Shahriar, Charles German

**Affiliations:** a The University of Chicago Medical Center, Chicago, Illinois, USA; b Georgetown University, Washington, DC, USA

We were intrigued by the recent observational study by Kamel et al^1^ examining the relationship between cannabis use and cardiovascular (CV) outcomes among relatively healthy young adults in the United States. This was a large, retrospective analysis of electronic medical record data across 53 health organizations in the United States. Their work provides a timely addition to the CV public health literature given the widespread legalization of recreational cannabis use. Given the relatively large effect sizes and limited space for discussion under the brief report format, we kindly ask the authors to address the following points to aid with interpretation of their findings:

First, the prevalence of cannabis use in their study was 2%. The cannabis-user group included those with any of the following ICD-10 diagnostic codes: F12.1 (cannabis abuse), F12.9 (cannabis use), and F12.90 (cannabis use, unspecified). In nationally representative survey research conducted over the same period, cannabis use among young adults (aged 19-55 years) is much more common: 26.9% for any 12-month use, 16.9% for any 30-day use, and 6.2% for near-daily use.^2^ Likewise, the prevalence of 12-month and lifetime cannabis use disorder are 2.5% and 6.3%, respectively.^3^ Our suspicion is that the low prevalence in this study is due to the generally poor sensitivity of ICD-10 codes as a proxy for substance use.^4^ Another possible explanation is the exclusion of those with tobacco use, which carries significant overlap.^3^ We would appreciate the authors’ input on the discrepancy of an order of magnitude in prevalence.

Second, cannabis use disorder is often comorbid with other substance use disorders (SUDs) and psychiatric conditions.^3^ The authors excluded patients with comorbid tobacco use, but there was no mention of alcohol use disorder, opioid use disorder, or abuse of cocaine or amphetamines—many of which are also associated with excess CV risk. Could the authors provide rationale for the omission of these other SUDs or provide their prevalences in both study groups?

To tie these points together, if only severe cases of cannabis use are being captured in the “cannabis-user” cohort, then until proven otherwise, it is reasonable to assume an even higher prevalence and severity of comorbid SUDs. These results should be interpreted with caution due to the possibilities of both misclassification bias and residual confounding.

## References

[1] Kamel I., Mahmoud A.K., Twayana A.R., Younes A.M., Horn B., Dietzius H. (2025). Myocardial infarction and cardiovascular risks associated with cannabis use: a multicenter retrospective study. JACC Adv.

[2] Patrick M.E., Pang Y.C., Terry-McElrath Y.M., Arterberry B.J. (2024). Historical trends in cannabis use among U.S. Adults ages 19–55, 2013–2021. J Stud Alcohol Drugs.

[3] Hasin D.S., Kerridge B.T., Saha T.D. (2016). Prevalence and correlates of DSM-5 cannabis use disorder, 2012-2013: findings from the national epidemiologic survey on alcohol and related conditions-III. Am J Psychiatry.

[4] McGrew K.M., Homco J.B., Garwe T. (2020). Validity of International classification of diseases codes in identifying illicit drug use target conditions using medical record data as a reference standard: a systematic review. Drug Alcohol Depend.

